# Neural Tracking of Sound Rhythms Correlates With Diagnosis, Severity, and Prognosis of Disorders of Consciousness

**DOI:** 10.3389/fnins.2021.646543

**Published:** 2021-04-28

**Authors:** Chuan Xu, Jiajie Zou, Fangping He, Xinrui Wen, Jingqi Li, Jian Gao, Nai Ding, Benyan Luo

**Affiliations:** ^1^Department of Neurology, First Affiliated Hospital, School of Medicine, Zhejiang University, Hangzhou, China; ^2^Key Laboratory for Biomedical Engineering of Ministry of Education, College of Biomedical Engineering and Instrument Sciences, Zhejiang University, Hangzhou, China; ^3^Research Center for Advanced Artificial Intelligence Theory Zhejiang Lab, Hangzhou, China; ^4^Department of Rehabilitation, Hangzhou Mingzhou Brain Rehabilitation Hospital, Hangzhou, China

**Keywords:** disorders of consciousness, neural synchronization, auditory steady state response, EEG, machine learning

## Abstract

Effective diagnosis and prognosis of patients with disorders of consciousness (DOC) provides a basis for family counseling, decision-making, and the design of rehabilitation programs. However, effective and objective bedside evaluation is a challenging problem. In this study, we explored electroencephalography (EEG) response tracking sound rhythms as potential neural markers for DOC evaluation. We analyzed the responses to natural speech and tones modulated at 2 and 41 Hz. At the population level, patients with positive outcomes (DOC-P) showed higher cortical synchronization to modulated tones at 41 Hz compared with patients with negative outcomes (DOC-N). At the individual level, phase coherence to modulated tones at 41 Hz was significantly correlated with Coma Recovery Scale-Revised (CRS-R) and Glasgow Outcome Scale-Extended (GOS-E) scores. Furthermore, SVM classifiers, trained using phase coherences in higher frequency bands or combination of the low frequency aSSR and speech tracking responses, performed very well in diagnosis and prognosis of DOC. These findings show that EEG response to auditory rhythms is a potential tool for diagnosis, severity, and prognosis of DOC.

## Introduction

Prolonged disorders of consciousness (DOC) is a group of neurological syndromes caused by severe brain damage, in which impairment of consciousness lasts more than 28 days from onset (Giacino et al., 2018). Prolonged DOC patients include patients in vegetative state (VS)/unresponsive wakefulness syndrome (UWS) and patients in minimally conscious state (MCS). VS/UWS is a condition of wakefulness without awareness (Laureys et al., 2010). These patients may have their eyes open but exhibit reflex behaviors only; therefore, they are considered unaware of themselves and their surroundings (Naccache, 2018). On the contrary, MCS patients show unequivocal signs of non-reflex cortically mediated behaviors in response to environmental stimuli, which occur inconsistently but are reproducible (Giacino et al., 2002). The Coma Recovery Scale-Revised (CRS-R) is a behavioral assessment method recommended to determine the level of consciousness in DOC (Estraneo et al., 2015; Iazeva et al., 2018; Kondziella et al., 2020). However, determining consciousness in unresponsive patients by behavioral assessment is challenging because patients must be awake during evaluation. In addition, patients must possess the voluntary drive to mobilize motor function. This trait must be preserved to a degree that is readily measurable (Kondziella et al., 2020). Furthermore, origin of most clinical signs and behaviors in DOC has not been fully explored and their correlation with patient consciousness is not fully known, making the method unreliable (Schnakers et al., 2009; Cortese et al., 2015; van Erp et al., 2015). These limitations and unavailability of adequate diagnostic tools result in approximately 40% misdiagnosis cases in DOC (Stender et al., 2014).

Several neuroimaging technologies have been proposed for assessing brain activity, such as MRI (Bardin et al., 2011; Monti et al., 2015; Edlow et al., 2017), PET (Stender et al., 2014, 2016), and fNIRS (Kurz et al., 2018; Abdalmalak et al., 2020) to circumvent the limitations of traditional diagnostic methods. Previous studies recommend integration of standardized clinical evaluation, electroencephalography (EEG)-based techniques, and functional neuroimaging to achieve multimodal evaluation of DOC (Kondziella et al., 2020). However, functional neuroimaging is not widely available and it is not clinically feasible for use in large numbers of patients. EEG is an attractive option as it is portable, cost-effective, and relatively feasible to deploy at the patient’s bedside (Chennu et al., 2017). Recent cross-sectional studies report that the brain networks, based on bedside high density electroencephalography (hdEEG) at rest, can be used to predict brain metabolic changes, diagnosis, and prognosis of DOC (Chennu et al., 2014, 2017; Wu et al., 2020). A previous longitudinal study reports that functional brain networks based on bedside hdEEG are an important prognosis predictor for DOC (Bareham et al., 2020). Moreover, the auditory Steady State Response (aSSR) is a promising tool for diagnosis and prognosis of DOC (Gorska and Binder, 2019).

When listening to sound with temporal modulations, neural activity can synchronize to the modulations. In human EEG and MEG recordings, such neural synchronization is most salient in two frequency bands. One frequency occurs around 40 Hz (Ross et al., 2000) and the other frequency occurs below 10 Hz (Wang et al., 2012). Neural synchronization to temporal modulations around 40 Hz is observed when listening to tones amplitude or frequency modulated at 40 Hz (Luo et al., 2006; Ding and Simon, 2009; Millman et al., 2010), and such responses are usually referred as the 40 Hz aSSR (Galambos et al., 1981). Neural synchronization to slow modulations below 10 Hz is observed when listening to tones modulated at a slow rate or when listening to natural speech (Ding and Simon, 2012b; Peelle et al., 2013; Doelling et al., 2014; Harding et al., 2019). Previous studies report that the phase-locking index of 40 Hz aSSR is an indicator of the level of central nervous system dysfunction in DOC (Binder et al., 2017, 2020). Further, previous studies report that neural response is observed in the sideband which reflects interaction between AM and FM response (Luo et al., 2006). In this study, we analyzed the response at 40 Hz and in sideband at 41 ± 2 Hz. Similar to high-frequency stimulus-synchronized responses to modulated tones, it is also found that neural synchronization at low rates can be used as an objective estimate of the level of neural dysfunction in DOC patients (Gorska and Binder, 2019).

Previous studies report that neural synchronization to speech is mainly observed in delta and theta bands for language conditions (Luo et al., 2006; Ding and Simon, 2012b, 2014). Notably, theta-band neural response and delta-band neural response plays different functions. For instance, theta-band response encodes syllabic-level acoustic features critical for speech recognition, whereas delta-band response is related to the perceived non-speech-specific acoustic rhythm (Ding and Simon, 2014). Studies have also explored speech tracking responses in DOC and show that speech tracking response is progressively delayed across healthy individuals, MCS patients, and UWS patients (Braiman et al., 2018). In this study, we explored the combination of multiple passive EEG paradigms, including neural synchronization to temporal modulations and speech tracking responses, to improve diagnosis and prognosis of DOC.

Herein, we recorded the 40 Hz aSSR, the low-frequency stimulus-synchronized responses to modulated tones, and speech tracking responses in the same groups of participants, aimed at elaborating the clinical utility of the three responses in DOC. In addition, we analyzed the potential of the three responses in diagnosis and prognosis of DOC.

## Materials and Methods

### Subjects and Neurobehavioral Assessments

A total of 47 subjects were included in this study. The subjects included 16 healthy individuals, 15 MCS patients, and 16 UWS patients. Five CRS-R assessments were performed in DOC patients 10 days before EEG recording. Diagnosis of the patients was based on the highest score of five CRS-R assessments (Wannez et al., 2017; Kondziella et al., 2020). Detailed information on patients, including sex, age, etiology, diagnosis, CRS-R results, time after the injury, and GOS-E results are presented in Supplementary Table 1. Two evaluations using CRS-R (before the experiment, 6 months later) and one evaluation using GOS-E (6 months after the experiment) were carried out and were also presented in Supplementary Table 1. The inclusion criteria for this study were as follows: (i) diagnosis with UWS, MCS based on the highest score of 5 assessments carried out for a period of 10 days by DOC experts using CRS-R (Kondziella et al., 2020); (ii) patients who had stayed for more than 1 month after brain injury; (iii) patients with no history of hearing impairment before brain injury; (iv) patients not under centrally acting drugs, neuromuscular function blockers, or sedation within 24 h prior to the study, patients should not have visible skull bone defects (CT); (v) patients with no history of neurodegenerative diseases such as Alzheimer’s disease and Parkinson’s disease before brain injury. Follow-up of behavioral measurements (CRS-R and GOS-E) of patients was carried out for more than 6 months after EEG assessments. An informed consent was acquired from their legal surrogates for all patients, whereas all health individuals signed written informed consent. The study was approved by the Ethical Committee of the First Affiliated Hospital of Zhejiang University and Hangzhou Mingzhou Brain Rehabilitation Hospital.

### Procedures

All participants listened to speech and modulated tones while EEG responses were recorded. Natural speech included two chapters from the novel *The Supernova Era* by Cixin Liu (Chapter 16: Fun country and Chapter 18: Sweet dream period). The story was narrated in Mandarin by a female speaker. Narration of the two chapters took 34 min and 25 min, respectively. The two chapters were presented sequentially (first Chapter 16, then Chapter 18). Modulated tones were sinusoidally frequency modulated at 2 Hz and sinusoidally amplitude modulated at 41 Hz. Speech and modulated tones were both presented binaurally through headphones. The experiment was performed in two separate days. All participants had their eyes open at the beginning of the experiment on each day. The spoken narrative was presented on the first day of the experiment. The modulated tones were presented after the spoken narrative. On the second day of the experiment, the same spoken narrative presented on day 1 was presented. The 59-min speech stimulus was repeated and therefore the total speech stimulus took approximately 2 h, which was longer compared with the stimulus duration in most studies. A long stimulus was used to effectively estimate the response phase. Healthy individuals and EMCS patients were asked to keep still during the experiment. No other tasks were allowed during the experiment; therefore, the participants listened passively. Data collection on the second day was terminated by the participant or the legal surrogate for three MCS patients and one healthy individual, therefore, for these participants we only analyzed the response to modulated tones.

### EEG Data Collection

Electroencephalography signals were recorded using a 64-electrodes BrainCap (Brain Products DmbH, Munich, Germany) in the international 10–20 system while listening to the auditory materials, and 1 of the 64 electrodes was placed under the right eye to record electrooculogram (EOG). EEG signals were referenced online to FCz, but were referenced offline to a common average reference. All signals were sampled at 1 kHz with a 50 Hz zero-phase Butterworth notch filter applied online.

### EEG Data Analysis

Electroencephalography recordings were low-pass filtered below 50 Hz with a zero-phase anti-aliasing FIR filter (implemented using a 200 ms Kaiser window) and down-sampled to 100 Hz. EOG artifacts were regressed out based on the least-squares method. Speech tracking EEG responses were expected to be similar across the two presentations of the same story; therefore, EEG recordings were averaged across recording days. Cerebro-acoustic phase coherence spectrum was calculated to characterize neural synchronization to auditory stimuli (modulated tones or speech) (Peelle et al., 2013). EEG response and acoustic envelope were segmented into non-overlapping 2 s time bins, and all segments were converted into the frequency domain using Discrete Fourier Transform (DFT). Cerebro-acoustic phase coherence was a function of frequency, and it quantified synchronization between sound envelope and neural response in each frequency bin. It was formulated as

$$C_{f}={\left(\sum_{t=1}^{T}\cos\left(\alpha_{f\,t}-\beta_{f\,t}\right)\right)}^{2}+{\left(\sum_{t=1}^{T}\sin\left(\alpha_{f\,t}-\beta_{f\,t}\right)\right)}^{2},$$

where *α_*ft*_* and *β_*ft*_* independently denote the response phase and stimulus phase in frequency bin *f* and time bin *t*. *C*_*f*_ is the phase coherence in frequency bin *f*. Higher phase coherence indicated that the response phase was more precisely synchronized to the stimulus phase. Based on the Rayleigh test for phase coherence (Fisher, 1993), if the *C*_*f*_ is larger than three, the phase distribution is significantly different from a uniform distribution (*P* < 0.05).

For the modulated tone, the period of the stimulus was 0.5 s. Therefore, in each 2 s time bin, the stimulus was identical and therefore *β_*ft*_* was a constant. In this case, the calculation of *C*_*f*_ reduced to

$$C_{f}={\left(\sum_{t=1}^{T}\cos\left(\alpha_{f\,t}\right)\right)}^{2}+{\left(\sum_{t=1}^{T}\sin\left(\alpha_{f\,t}\right)\right)}^{2},$$

which is similar to the inter-trial phase coherence (Luo and Poeppel, 2007). Phase coherence was strongest in central-frontal electrodes; therefore, 14 centro-frontal electrodes, i.e., Fz, F1, F2, F3, F4, FC1, FC2, FC3, FC4, Cz, C1, C2, C3, and C4, were used to compare the phase coherence value across populations and to perform correlation analysis and SVM classification.

### Response Topography

Phase coherence was always positive and measured how strongly the response phase was synchronized to the stimulus phase. However, when analyzing the response topography, it was useful to show whether the responses in different EEG electrodes were in phase or out of phase. Therefore, a signed phase coherence, a simplified version of the complex-valued topography, was considered in the response topography analysis (Simon and Wang, 2005). Specifically, channel Fz was chosen as a reference since it showed strongest phase coherence in most conditions. For each electrode, if the phase difference between this electrode and electrode Fz was larger than 90°, the phase coherence was negated. On the contrary, if the phase difference between this electrode and electrode Fz was less than 90°, the phase coherence was kept positive. The signed phase coherence can illustrate the phase relationship between electrodes on top of showing the phase coherence.

### Statistical and Classification Analysis

To evaluate whether response phase coherence at one frequency was significantly higher than chance, we first estimated the chance-level distribution of phase coherence.

In the responses to modulated tones, since responses were expected at integer frequencies, the responses at non-integer frequencies, i.e., 0.5, 1.5, …, 49.5 Hz, were considered as chance-level phase coherence. Furthermore, the test was applied to the group response; therefore, if the group included N participants, we estimated the chance-level distribution by randomly selecting N participants from all 47 participants and averaged the responses over the N participants. This sampling procedure was repeated 1,000 times. The phase coherence from the 1,000 times at the 50 non-integer frequencies were pooled. If the response coherence at one frequency was lower than the Ns of the 50,000 chance-level coherence values (one-sided comparison), its significance value was calculated as Ns/50,000. This procedure was used instead of the Rayleigh test to evaluate the significance of phase coherence since the phase coherence was averaged over channels and participants, whereas the Rayleigh test applies to data in a single channel from a single participant.

**FIGURE 1:**
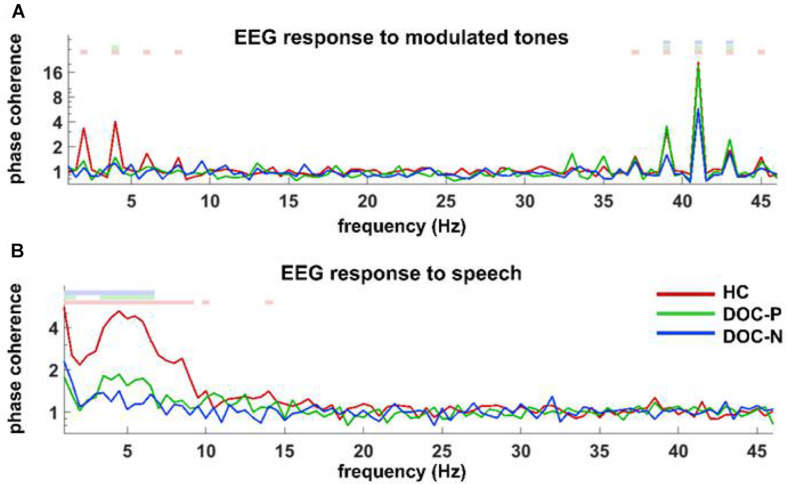
Phase coherence spectrum for responses to modulated tones **(A)** and speech **(B)** in the group of healthy controls (HC), patients with positive prognosis (DOC-P), and patients with negative prognosis (DOC-N). **(A)** Coherences to modulated tones at 41 and 41 ± 2 Hz were significant in HC, DOC-P, DOC-N, and there seemed to be no difference between HC and DOC-P. **(B)** Speech tracking response in delta and theta frequency bands for the group of healthy controls (HC) was significantly different from that of DOC-P group. The colored squares on top denote the frequency bins in which phase coherence is significantly higher compared with chance (*P* < 0.01, bootstrap, FDR corrected).

Bias-corrected and accelerated bootstrap was used for the unpaired comparisons between groups in Figure 1 (Efron and Tibshirani, 1994). Data from each group were resampled 5,000 times with replacement. This comparison was also one-sided. If in N out of 5,000 times, the mean in one group was greater (or smaller) than the other group, the significance level was (*N* + 1)/5,001. Correlational analyses were performed using two-tailed bivariate Pearson correlation coefficient. In this study, correlation analysis was performed between CRS-R score, GOS-E score, and the phase coherence. The single outlier was removed from correlation analysis to ensure reliability of the results.

Two-class support vector machine (SVM) classifiers were trained by phase coherence to modulated tones and natural speech to determine whether the neural synchronization to fast and slow auditory rhythms are potential application in diagnosis and prognosis of DOC. SVM is a classic machine-learning model used for classification using both small and large sample sizes (Way et al., 2010). For structural dependence models SVM, the special principle and algorithm is to find a hyper-plane in high dimensional space with feature vectors from the samples. Therefore, SVM was used in this study as an effective prediction model for classification of variables using the limited sample (Zhang et al., 2018). Radial basis function (RBF) was used as the SVM kernel function, which is the most common kernel function used to map data into a space (Gromski et al., 2014). Samples were divided randomly into training and test sets to determine the generalization of the model. Approximately 70% of UWS and MCS patients were randomly selected as training sets to build the model. A grid search method within a five-fold cross-validation procedure was used to optimize the regularization parameter of the SVM model (Cost and γ). The remaining 30% of patients were used as a test set to validate the function. Phase coherence at 41 and 41 ± 2 Hz, phase coherence at 2 and 4 Hz, phase coherence for the speech tracking response in delta and theta bands, combination of the phase coherence at 2 and 4 Hz, phase coherence to natural speech in delta, theta bands were used to train the SVM classifiers. Phase coherence at 41 and 41 ± 2 Hz, phase coherence at 2 and 4 Hz from 31 patients including of 15 MCS patients and 16 UWS patients were used in DOC classification analysis. The EEG data of the speech tracking response of three MCS patients were incomplete; therefore, phase coherence for the speech tracking response in delta and theta bands, the combination of phase coherence at 2 and 4 Hz, phase coherence for speech tracking response in delta, theta bands for 28 patients, including 12 MCS patients and 16 UWS patients, were used in the DOC classification analysis.

Follow-up of behavioral measurements (CRS-R and GOS-E) of patients was carried out for more than 6 months after EEG assessments for prediction of patient outcome. Each patient was initially labeled as showing a positive or negative outcome. Clinical diagnoses of patients were subcategorized into four different subclasses based on proposed ascending levels of consciousness, namely UWS, MCS−, MCS+, and EMCS. In this study, positive outcome was defined as any advances in transition of clinical categorization during follow-up, whereas negative outcome was defined as stasis or retrogress in transition. Phase coherence at 41 and 41 ± 2 Hz, phase coherence at 2 and 4 Hz of 31 patients including 17 patients with positive outcomes and 14 patients with negative outcomes were used in prognosis prediction analysis. Three MCS patients were excluded due to incomplete EEG data of the speech tracking response. Therefore, phase coherence for the speech tracking response in delta and theta bands, combination of phase coherence at 2 and 4 Hz, phase coherence for the speech tracking response in delta, theta bands of 28 patients, including 15 patients with positive outcomes and 13 patients with negative outcomes were used in prognosis prediction analysis. Diagnosis and prediction analysis in this study were conducted using the python program.

## Results

### Neural Synchronization to Auditory Rhythms in DOC With Good and Poor Prognosis

**FIGURE 2:**
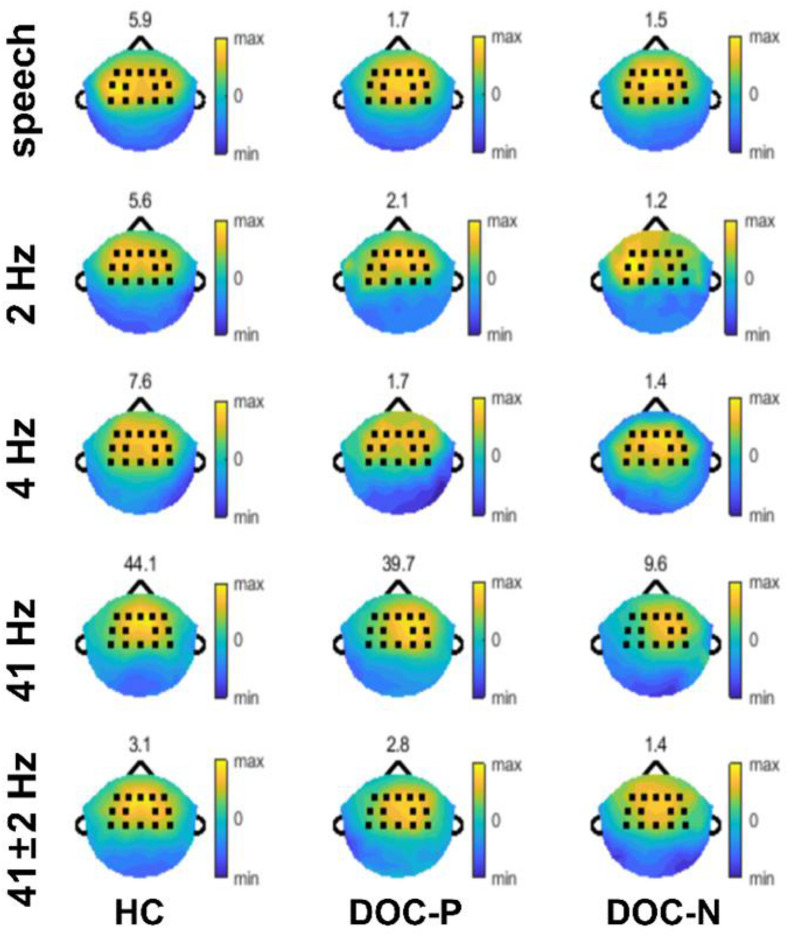
Topography of phase coherence. Phase coherence was separately normalized for each population by dividing by the 95th percentile of phase coherence across electrodes, to accurately illustrate the spatial distribution, and the values of the 95th percentile are shown on top of each plot.

**FIGURE 3:**
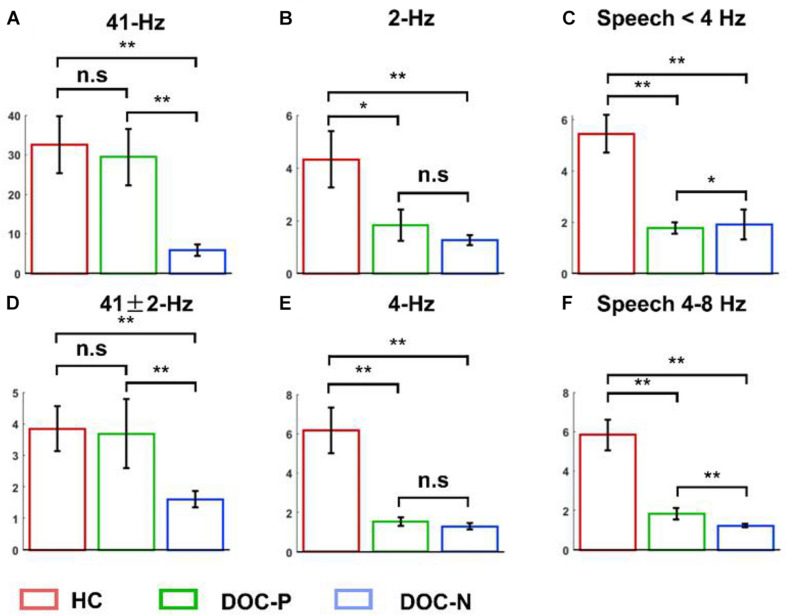
Mean and SEM of the phase coherence between neural responses and acoustic envelope in the healthy controls (HC), patients with positive prognosis (DOC-P), and patients with negative prognosis (DOC-N). **(A,D)** There were no statistically significant differences in the 41 and 41 ± 2 Hz phase coherences between for HC and DOC-P. However, the 41 and 41 ± 2 Hz phase coherences were significantly higher in HC and DOC-P groups compared with those for DOC-N group. **(B,E)** The 2 and 4 Hz phase coherences in HC were significantly higher compared with those for DOC-P. **(C,F)** Phase coherences to natural speech in theta and delta bands were significantly different between HC and DOC-P groups (*P < 0.05, **P < 0.01; n.s., not significant).

To explore neural synchronization to auditory rhythms in DOC with good and poor prognosis, patients with positive prognosis (DOC-P) were considered as a separate group, patients with poor prognosis (DOC-N) were grouped as another separate group, then phase coherence between neural responses and acoustic envelope in the group of healthy controls (HC), DOC-P, and DOC-N was calculated. The results in Figure 1 were averaged across all electrodes. As shown in Figure 1, phase coherences to modulated tones at 41 and 41 ± 2 Hz were significant in HC, DOC-P, and DOC-N, and there seemed to be no difference for phase coherences to modulated tones at 41 and 41 ± 2 Hz between HC and DOC-P. However, the difference of speech tracking response in delta and theta bands between the group of healthy controls (HC) and DOC-P were observed. Analysis of topography of the neural synchronization to auditory rhythms showed a centro-frontal distribution for all 3 groups of participants (Figure 2). Therefore, 14 centro-frontal channels were selected for further analyses. Phase coherence averaged across centro-frontal electrodes were compared across populations as shown in Figure 3. Phase coherence to modulated tones at 41 and 41 ± 2 Hz for HC group was not significantly different compared with that of the DOC-P group. However, phase coherences at 41 and at 41 ± 2 Hz in the HC and DOC-P groups were significantly higher compared with that in the DOC-N group (*P* < 0.01, bootstrap, FDR corrected) (Figures 3A,D). Phase coherences for DOC-P at 2 and 4 Hz were not significantly different compared with those for DOC-N group (*P* > 0.05, bootstrap, FDR corrected) (Figures 3B,E). For speech tracking response, phase coherences to natural speech in theta and delta bands for HC group were significantly different compared with those for DOC-P group (*P* < 0.01, bootstrap, FDR corrected) (Figures 3C,F).

### Correlations Between Neural Synchronization and the Clinical Behaviors

**FIGURE 4:**
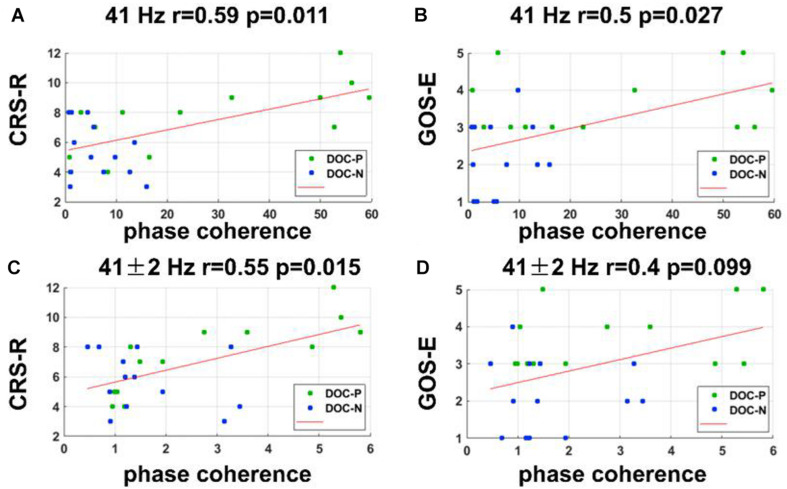
Correlations between neural synchronization at 41, 41 ± 2 Hz, and clinical evaluations. **(A,B)** Correlations between the phase coherence at 41 Hz and CRS-R, and GOS-E scores. Phase coherence at 41 Hz was independently correlated with CRS-R and GOS-E scores (*r* = 0.59, *P* = 0.011, FDR corrected; *r* = 0.5, *P* = 0.027, FDR corrected). **(C,D)** Correlations between the phase coherence at 41 ± 2 Hz and CRS-R, and GOS-E scores. Phase coherence at 41 ± 2 Hz was correlated with CRS-R score (*r* = 0.55, *P* = 0.015, FDR corrected).

**FIGURE 5:**
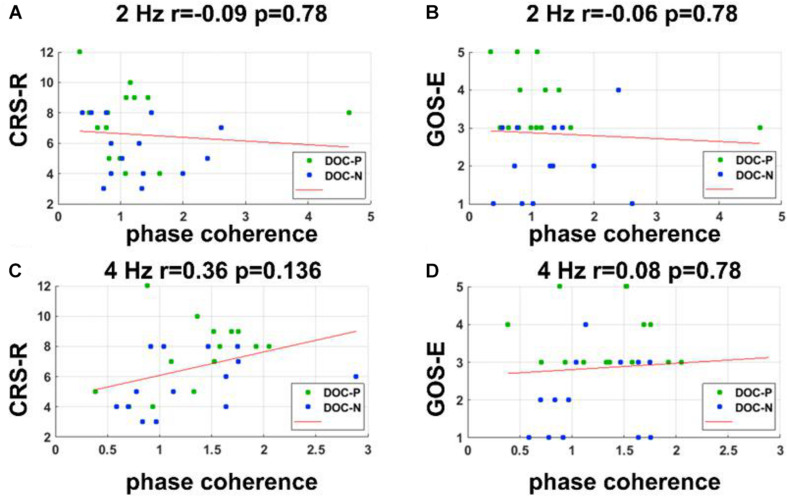
Correlations between neural synchronization at 2 and 4 Hz, and clinical evaluations. **(A–D)** Correlations between phase coherence at 2, 4 Hz, and CRS-R, GOS-E scores. Correlations between the phase coherence at 2, 4 Hz, and CRS-R, GOS-E were not statistically significant.

**FIGURE 6:**
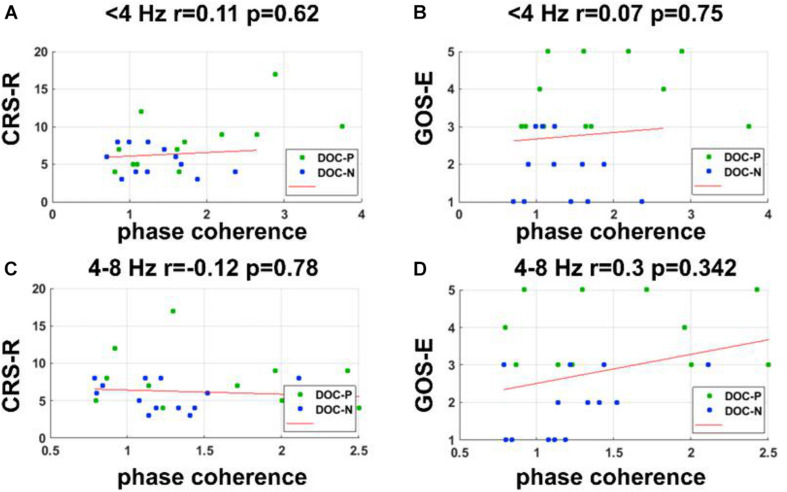
Correlations between the phase coherence for the speech tracking response in delta and theta bands and clinical evaluations. **(A–D)** Correlations between the phase coherence for the speech tracking response in delta, theta bands, and CRS-R, GOS-E score showed no statistical significance.

To evaluate whether neural synchronization to temporal modulations and natural speech had values to determine the diagnosis, severity, and prognosis of DOC, we set out to assess the correlations between the neural synchronization and clinical assessments of neurological function in individual patients. A single outlier was removed before performing correlation analysis to ensure the reliability of the results. Phase coherence at 41 Hz was independently correlated with CRS-R and GOS-E (*r* = 0.59, *P* = 0.011, FDR corrected; *r* = 0.5, *P* = 0.027, FDR corrected) (Figure 4). Furthermore, phase coherence at 41 ± 2 Hz was correlated with CRS-R (*r* = 0.55, *P* = 0.015, FDR corrected) (Figure 4). Analysis showed that correlations between the phase coherence at 2 and 4 Hz, CRS-R, and GOS-E were not significant (Figure 5). In addition, correlation between phase coherence to natural speech in lower frequency bands and CRS-R and correlation between phase coherence to natural speech in lower frequency bands and GOS-E were not significant (Figure 6). Details on correlation coefficient analysis between CRS-R total score, GOS-E total score, and cerebro-acoustic phase coherence are shown in Table 1.

**TABLE 1:** Correlations between CRS-R total score, GOS-E total score, and cerebro-acoustic phase coherence (FDR corrected). Significant *P*-values (<0.05) are shown in bold.

	**41-Hz**		**41 ± 2-Hz**		**2 Hz**		**4 Hz**		**Speech <4 Hz**		**Speech 4–8 Hz**	
	***r***	***p***	***r***	***p***	***r***	***p***	***r***	***p***	***r***	***p***	***r***	***p***
Total CRS-R score	0.59	**0.011**	0.55	**0.015**	−0.09	0.78	0.36	0.136	0.11	0.62	−0.12	0.78
Total GOS-E score	0.5	**0.027**	0.4	0.099	−0.06	0.78	0.08	0.78	0.07	0.75	0.3	0.342

### Classification and Prognosis Prediction Based on Neural Synchronization in DOC

To further analyze the role of neural synchronization in diagnosis and prognosis of DOC, two-class SVM classifiers were trained using the multiple EEG measurements. SVM classifiers were trained separately using phase coherences at 41 and 41 ± 2 Hz, phase coherences at 2 and 4 Hz, phase coherences for speech tracking response in delta and theta bands, combination of phase coherences at 2 and 4 Hz, and phase coherences for the speech tracking response in delta, theta bands.

**FIGURE 7:**
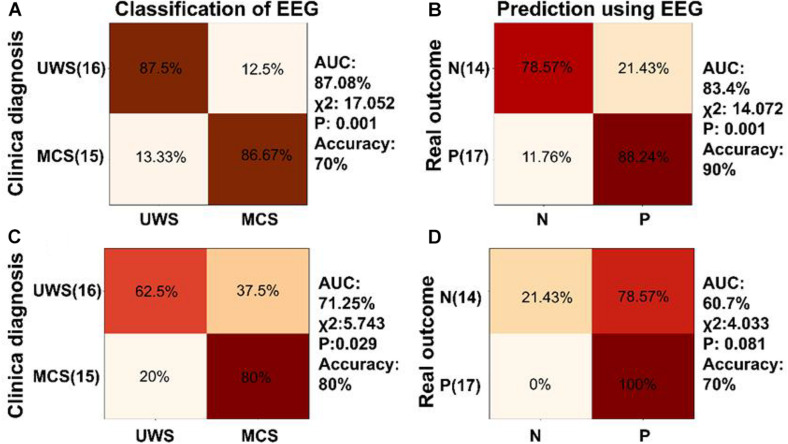
Classification and prognosis prediction in DOC using neural synchronization to temporal modulations at low and high frequencies. **(A)** Diagnosis classification using phase coherence at 41 and 41 ± 2 Hz performed well (AUC = 87.08%, accuracy = 70%, χ^2^ = 17.052, *P* = 0.001). Confusion matrix generated by SVM showed 86.67% sensitivity and 87.5% specificity for MCS diagnosis. **(B)** The SVM classifier trained using phase coherence at 41 and 41 ± 2 Hz showed good performance in prognosis prediction of DOC (AUC = 83.4%, accuracy = 90%, χ2 = 14.072, *P* = 0.001) and was able to predict positive prognosis of individual patients with high sensitivity (88.24%) and specificity (78.57%). **(C,D)** The SVM classifier showed poor performance in classification and prognosis prediction of DOC trained using phase coherence at 2 and 4 Hz.

SVM classifiers were trained by phase coherences to modulated tones at 41 and 41 ± 2 Hz (Figures 7A,B). The confusion matrix showed that the classifier trained by phase coherence at 41 and 41 ± 2 Hz showed 86.67% sensitivity and 87.5% specificity in discriminating MCS and UWS (χ^2^ = 17.052, *P* = 0.001, accuracy = 70%, AUC = 87.08%, Figure 7A). In prognosis prediction of DOC, the SVM classifier trained by phase coherences at 41 and 41 ± 2 Hz performed very well (χ^2^ = 14.072, *P* = 0.001, accuracy = 90%, AUC = 83.4%) with high sensitivity (88.24%) and specificity (78.57%) in positive prognosis prediction (Figure 7B). However, SVM classifiers trained by phase coherence at 2 and 4 Hz did not perform well in classification and prognosis prediction of DOC (Figures 7C,D).

**FIGURE 8:**
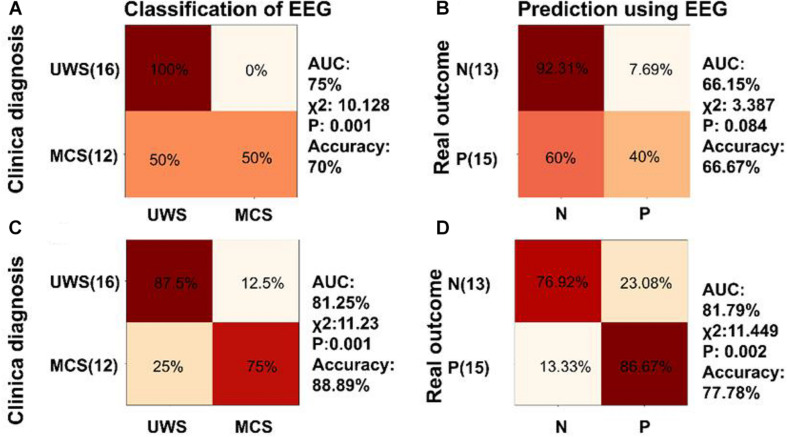
Classification and diagnosis prediction using speech tracking response and combination of low frequency aSSR and speech tracking responses. **(A,B)** Confusion matrix generated by the SVM classifier trained by the phase coherence to the natural speech in delta and theta bands for classification and prognosis prediction of DOC. **(C,D)** Confusion matrix generated by the SVM classifier trained by the combination of the phase coherence to modulated tones at 2, 4 Hz, and the phase coherence to natural speech in delta, theta bands for classification and prognosis prediction of DOC. The SVM classifiers showed better performance in classification and prognosis prediction of DOC compared with SVM classifiers separately trained with phase coherence at 2 and 4 Hz or the phase coherence to natural speech in delta and theta bands.

A two-class SVM classifier was trained by phase coherence to natural speech in delta and theta bands to analyze the role of speech tracking response in classification and prognosis prediction of DOC. The inputs to the classifier were delta and theta speech tracking response. The confusion matrix in Figure 8A, generated by the SVM classifier trained by the phase coherence to the natural speech in delta and theta bands, showed that the classifier had 50% sensitivity and 100% specificity in discriminating MCS and UWS (χ^2^ = 10.182, *P* = 0.001, accuracy = 70%, AUC = 75%, Figure 8A). However, this classifier did not perform well in prognosis prediction (χ^2^ = 3.387, *P* = 0.084, accuracy = 66.67%, AUC = 66.15%, Figure 8B).

Further, the classifier was trained using a combination of phase coherences at 2 and 4 Hz, and phase coherences for the speech tracking response in delta, theta bands. Analysis of the classifier showed 75% sensitivity and 87.5% specificity in discriminating MCS and VS (χ^2^ = 11.23, *P* = 0.001, accuracy = 88.89%, AUC = 81.25%, Figure 8C). In addition, the SVM classifier predicted positive prognosis of the individual patients with high sensitivity (86.67%) and specificity (76.92%) (χ^2^ = 11.449, *P* = 0.002, accuracy = 77.78%, AUC = 81.79%, Figure 8D). The performance of the SVM classifiers in classification and prognosis prediction of DOC was significantly higher using combined training of phase coherences at 2 and 4 Hz, and phase coherences for speech tracking response in delta, theta bands, compared with training using separate parameters.

## Discussion

Physical and cognitive impairments experienced by individuals with DOC vary greatly, and it is difficult to distinguish behaviors that are indicative of conscious awareness from those that are random and non-purposeful, making diagnosis and prognosis challenging. Previous studies report that multimodal evaluations have high sensitivity in detection of consciousness (Giacino et al., 2018). In this study, the role of multiple passive EEG paradigms in the diagnosis and prognosis of DOC was explored. The neural synchronization to auditory rhythms in DOC patients with good and poor prognosis was analyzed. Phase coherences at 41 and 41 ± 2 Hz in DOC-N were significantly lower compared with those of HC and DOC-P. However, phase coherences at 41 and 41 ± 2 Hz of HC were not significantly different from those of DOC-P group. This finding implies that DOC patients with normal aSSR in higher frequency bands may retain specific aspects of cerebral function related to prognosis. Further, correlation analysis showed that neural synchronization to modulated tones at 41 Hz in DOC was correlated with GOS-E, the scoring scale used for prognosis of DOC. Moreover, SVM classifier was trained using phase coherence at 41 and 41 ± 2 Hz. Analysis of the results showed that the classifier predicted positive prognosis of individual patients with high sensitivity and specificity. For speech tracking response, SVM classifier was trained using delta and theta speech tracking response and analysis showed that the classifier accurately classified MCS and UWS. Notably, SVM classifiers trained using a combination of phase coherences at 2 and 4 Hz, and phase coherences for the speech tracking response in delta, theta bands showed better performance in classification and prognosis prediction of DOC, compared with training using the separate parameters.

The 40 Hz aSSR and low-frequency auditory responses below 10 Hz reflect neural processing on different time scales and are likely to be generated from different neural sources. The 40 Hz aSSR belongs to the gamma band and is generated in primary auditory cortex and auditory midbrain and thalamus (Ross et al., 2005; Steinmann and Gutschalk, 2011). Previous studies report that progressive deafferentation in neurons within the central thalamus is in proportion to the severity of structural brain injuries, and central thalamus plays a crucial role in the maintenance of consciousness (Maxwell et al., 2006; Giacino et al., 2014). Therefore, DOC patients can be classified by detecting 40 Hz aSSR that reflects thalamic function. Previous studies also report that 40 Hz aSSR is positively correlated with CRS-R total score and with the scores of the Auditory and Visual subscales in DOC (Binder et al., 2017, 2020). In this study, analysis showed that 40 Hz aSSR was correlated with CRS-R total score. Moreover, the 40 Hz aSSR was positively correlated with the GOS-E score. Furthermore, the SVM classifier trained using the aSSR at 41 and 41 ± 2 Hz, accurately classified MCS and UWS and predicted the good and poor prognosis in DOC. These findings imply that aSSR at high frequency has broad application prospects in diagnosis and prognosis of DOC. Low-frequency stimulus-synchronized responses and speech tracking responses in delta and theta bands are generated from more broad cortical areas in the temporal and frontal lobes (Ding and Simon, 2012a; Zion Golumbic et al., 2013). In this study, low-frequency stimulus-synchronized responses to modulated tones showed no value in diagnosis and prognosis of DOC.

Recent studies show that speech tracking responses are progressively delayed along with the awareness decline in healthy individuals, MCS patients, and UWS patients, and may help to identify cognitive motor dissociation (CMD) in DOC (Braiman et al., 2018). In this study, SVM classifier was trained using delta and theta speech tracking response. Analysis showed that the classifier accurately distinguished MCS from UWS. A recent study using isochronously presented speech reports that EEG-derived neural signals, including speech-tracking responses and temporal dynamics of global brain states, are associated with behavioral diagnosis of consciousness and are accurate in prediction of future outcomes in individual patients (Gui et al., 2020). In this study, analysis showed that speech tracking response is a potential indicator for distinguishing MCS from UWS.

In this study, a passive paradigm was used to explore whether neural tracking of sound rhythms is useful in determining residual consciousness in DOC. Compared with passive paradigms, cognitive demands of active paradigms are higher. Although successful demonstration of covert command-following is a widely accepted clinical marker of awareness and useful for prognosis, its sensitivity is compromised by precluding many patients with cognitive deficits from demonstrating the extent of their abilities (Guger et al., 2003; Monti et al., 2010). Furthermore, passive paradigm is more convenient and practical in clinical applications compared with active paradigm. A positive response to stimulation is covert cortical processing in passive paradigms. Notably, ability of covert cortical processing to reflect residual consciousness process depends on the types of passive paradigm. However, a positive response in active paradigms may reflect CMD.

A number of active paradigm studies report that patients with severe brain injury may not reveal any signs of consciousness at the bedside, but some of them are able to willfully modulate their brain activity on command, even occasionally answering yes/no questions by performing mental imagery tasks (Monti et al., 2010). Approximately 15% of behaviorally VS/UWS patients are able to follow commands by modifying their brain activity during an EEG- and/or fMRI-based active consciousness paradigm, implying that they have covert cognitive abilities (Kondziella et al., 2016). CMD defines patients who demonstrate sharp dissociation of an inability or extremely limited ability to move with preservation of higher-level cognition in the form of reliable command-following, as detected with functional MRI, EEG, or other non-invasive measures (Schiff, 2015). Emerging evidence indicates that CMD patients represent a distinct subgroup of patients with DOC, whose brain networks and clinical features might fundamentally differ from those of other subgroups (Pincherle et al., 2019; Edlow et al., 2020; Johr et al., 2020). Pan et al. (2020) report that patients with CMD have a better outcome compared with other patients (Pan et al., 2020). A previous study reported that cortical response to the natural speech envelope can help identify CMD (Braiman et al., 2018). CMD patients should be differentiated from DOC patients for determination of the application of various paradigms in diagnosis and prognosis in DOC more objectively and effectively in the future.

Currently, multimodal evaluation of DOC, integrated with EEG-based techniques and functional neuroimaging, is recommended as it is highly accurate (Kondziella et al., 2020). Approaches for detecting consciousness by means of positron emission tomography (PET), fMRI, and EEG have been developed in the past two decades to supplement clinical evaluation of DOC (Gosseries et al., 2014; Kondziella et al., 2016; Marino et al., 2016). The default mode network (DMN) is absent in brain death; however, it is partially preserved in VS, probably reflecting residual structural connectivity (Soddu et al., 2012). Several studies report the prognostic value of blood-oxygen-level-dependent (BOLD) signals elicited by a subject’s own name in traumatic VS (Wang et al., 2015). These studies report that functional neuroimaging has important implications for clinical diagnosis and prognosis for patients with disorders of consciousness. However, functional neuroimaging is not widely available and may not be clinically feasible in large numbers of patients. Combination of multiple passive EEG paradigms is more valuable for diagnosis and prognosis of DOC. The results in this study were consistent with the hypothesis: the performance of the SVM classifier trained by combination of phase coherence at 2 and 4 Hz, phase coherence for the speech tracking response in delta, theta bands performed better compared with the classifier separately trained using phase coherence at 2 and 4 Hz, or phase coherence for the speech tracking response in delta, theta bands. Multimodal evaluation based on a combination of multiple passive EEG paradigms has important implications and is more feasible for clinical diagnosis and prognosis of patients with disorders of consciousness.

However, this study had some limitations. In order to ensure the credibility of the research results, the inclusion criteria of subjects were relatively strict and prevalence rate of DOC was relatively low. These factors result in insufficient sample size in this study. To further verify these results, multi-center studies for neural synchronization to fast and slow auditory rhythms should be conducted in the future with larger sample sizes. Neural tracking of sound rhythms showed the potential in diagnosis and prognosis in DOC; however, the normal value range was not determined. In addition, heterogeneity of DOC etiology may affect the accuracy of the results. Further studies should be performed using patients with DOC caused by single etiology, such as traumatic brain injury.

In summary, analysis of phase coherences to natural speech and modulated tones at 2 and 41 Hz shows that EEG responses to auditory rhythms is a potential tool for predicting diagnosis, severity, and prognosis of DOC.

## Data Availability Statement

The original contributions presented in the study are included in the article/Supplementary Material, further inquiries can be directed to the corresponding author/s.

## Ethics Statement

The studies involving human participants were reviewed and approved by the Ethical Committee of the First Affiliated Hospital of Zhejiang University and Hangzhou Mingzhou Brain Rehabilitation Hospital. The patients/participants provided their written informed consent to participate in this study. Written informed consent was obtained from the individual(s) for the publication of any potentially identifiable images or data included in this article.

## Author Contributions

CX: investigation, data curation, and writing – review and editing. JZ: methodology, software, formal analysis, and writing – review and editing. FH: visualization and writing – review and editing. XW: visualization, resources, and writing – review and editing. JL and JG: investigation, resources, and data curation. ND: conceptualization, methodology, software, formal analysis, resources, writing – review and editing, and supervision. BL: conceptualization, resources, supervision, project administration, and writing – review and editing. All authors contributed to the article and approved the submitted version.

## Conflict of Interest

The authors declare that the research was conducted in the absence of any commercial or financial relationships that could be construed as a potential conflict of interest.
