# Supplementary material for: Neural Tracking of Sound Rhythms Correlates With Diagnosis, Severity, and Prognosis of Disorders of Consciousness
Source: Front Neurosci. 2021 Apr 28;15:646543. doi: 10.3389/fnins.2021.646543 (PMC8113690; doi:10.3389/fnins.2021.646543)
Supplement: Supplementary file 1 [file Table_1.DOC]

| **Patient** | **Aetiology** | **Gender/age** | **Time since**  **injury to EEG assessments (months)** | **Initial diagnosis** | **Highest CRS-R total score /sub-scores assessed before EEG assessments** | **Diagnosis assessed more than 6 months after EEG assessments** | **CRS-R total score /sub-scores assessed more than 6 months after EEG assessments** | **GOS-E assessed more than 6 months after EEG assessments** |
| --- | --- | --- | --- | --- | --- | --- | --- | --- |
| 1 | CVD | M/49 | 7 | UWS | 3/0-0-2-0-0-1 | UWS | 3/0-0-2-0-0-1 | 2 |
| 2^☆^ | CVD | M/48 | 3 | UWS | 4/0-0-2-1-0-1 | MCS- | 9/1-3-3-1-0-1 | 4 |
| 3^☆^ | CVD | M/40 | 4 | UWS | 5/0-1-2-1-0-1 | MCS- | 10/2-3-2-1-0-2 | 3 |
| 4^☆^ | ABI | M/47 | 3 | UWS | 8/2-1-2-1-0-2 | MCS- | 9/2-3-1-1-0-2 | 3 |
| 5 | CVD | F/73 | 4 | UWS | 7/1-1-2-1-0-2 | death | death | 1 |
| 6 | ABI | M/45 | 6 | UWS | 6/0-1-2-1-0-2 | UWS | 6/0-1-2-1-0-2 | 2 |
| 7 | TBI | F/73 | 3 | UWS | 4/0-0-2-1-0-1 | death | death | 1 |
| 8^☆^ | CVD | F/68 | 1 | UWS | 7/1-1-2-1-0-2 | MCS- | 9/1-3-2-1-0-2 | 3 |
| 9 | TBI | M/75 | 7 | UWS | 6/0-1-2-1-0-2 | death | death | 1 |
| 10 | TBI | M/38 | 5 | UWS | 4/0-0-2-1-0-1 | UWS | 7/112102 | 2 |
| 11^☆^ | CVD | M/52 | 1.5 | UWS | 7/1-1-2-1-0-2 | EMCS | 22/456232 | 5 |
| 12^☆^ | CVD | M/59 | 2 | UWS | 4/0-0-1-1-0-2 | MCS- | 12/1-3-5-1-0-2 | 3 |
| 13^☆^ | CVD | M/47 | 1.5 | UWS | 7/1-1-2-1-0-2 | MCS- | 13/2-3-5-1-0-2 | 3 |
| 14 | CVD | M/74 | 2.5 | UWS | 5/0-0-2-1-0-2 | death | death | 1 |
| 15 | CVD | M/60 | 2 | UWS | 3/0-0-1-1-0-1 | UWS | 7/2-2-1-1-0-1 | 2 |
| 16^☆^ | CVD | F/61 | 2 | UWS | 5/0-1-1-1-0-2 | MCS+ | 16/2-4-5-2-1-2 | 4 |
| 17 | CVD | M/38 | 8 | MCS+ | 8/0-3-2-1-0-2 | MCS+ | 8/0-3-2-1-0-2 | 3 |
| 18^☆^ | ABI | M/18 | 3 | MCS+ | 14/1-4-5-2-0-2 | EMCS | 20/4-5-6-2-1-2 | 5 |
| 19 | CVD | M/42 | 8 | MCS- | 8/0-3-2-1-0-2 | death | death | 1 |
| 20^☆^ | CVD | M/47 | 2 | MCS- | 9/0-1-5-1-0-2 | MCS+ | 17/3-5-5-1-1-2 | 4 |
| 21^☆^ | CVD | M/56 | 3 | MCS- | 9/2-3-1-1-0-2 | EMCS | 20/4-5-6-2-1-2 | 5 |
| 22^☆^ | CVD | M/63 | 4 | MCS+ | 12/1-3-5-1-0-2 | EMCS | 14/2-3-6-1-0-2 | 5 |
| 23 | TBI | M/36 | 3 | MCS- | 4/0-0-3-0-0-1 | MCS- | 8/1-2-3-1-0-1 | 3 |
| 24 | CVD | M/36 | 5 | MCS- | 8/0-3-2-1-0-2 | MCS- | 8/0-3-2-1-0-2 | 3 |
| 25^*☆^ | CVD | M/60 | 3 | MCS- | 9/0-1-5-1-0-2 | MCS+ | 16/2-4-5-2-1-2 | 4 |
| 26 | CVD | M/36 | 10 | MCS- | 8/0-3-2-1-0-2 | MCS- | 8/0-3-2-1-0-2 | 3 |
| 27^*☆^ | TBI | M/67 | 1.5 | MCS- | 5/0-0-3-1-0-1 | MCS+ | 17/4-5-5-1-0-2 | 4 |
| 28 | TBI | M/72 | 11.5 | MCS- | 10/1-1-5-1-0-2 | MCS+ | 12/2-2-5-1-0-2 | 3 |
| 29^☆^ | CVD | F/31 | 3.5 | MCS+ | 17/4-5-5-1-0-2 | EMCS | 21/4-5-6-2-1-3 | 5 |
| 30^*☆^ | CVD | M/67 | 1 | MCS- | 8/0-0-5-1-0-2 | MCS+ | 8/0-0-5-1-0-2 | 3 |
| 31^☆^ | TBI | M/67 | 2 | MCS- | 8/0-0-5-1-0-2 | MCS+ | 13/2-3-5-1-0-2 | 3 |

**Supplementary Table 1. The characteristic data of the patients in DOC.**

UWS, unresponsive wakefulness syndrome; MCS+, minimally conscious state plus; MCS-, minimally conscious state minus;CRS-R, Coma Recovery Scale-Revised; GOS-E, Glasgow Outcome Scale-Extended; ABI, anoxic brain injury; CVD, cerebrovascular disease; and TBI, traumatic brain injury. CRS-R sub-scores indicating the assessment of auditory, visual, motor, verbal, communication functions and arousal. * these patients, who couldn't complete the second speech tracking response. ^☆^ the patient with positive outcome.
